# Patient-reported outcomes in Primary Spinal Intradural Tumours: a systematic review

**DOI:** 10.1038/s41393-024-00987-6

**Published:** 2024-04-08

**Authors:** Ahmad M. S. Ali, Mohammad A. Mustafa, Omar M. E. Ali, Conor S. Gillespie, George M. Richardson, Simon Clark, Martin J. Wilby, Christopher P. Millward, Nisaharan Srikandarajah

**Affiliations:** 1 Department of Neurosurgery, The Walton Centre NHS Foundation Trust, Liverpool, UK; 2 School of Medicine, Liverpool University, Liverpool, UK; 3 Royal Victoria Infirmary, Newcastle upon-Tyne, UK; 4 Department of Clinical Neurosciences, University of Cambridge, Cambridge, UK; 5 Royal Liverpool University Hospital, Liverpool, UK; 6 Institute of Systems, Molecular, & Integrative Biology, University of Liverpool, Liverpool, UK

**Keywords:** CNS cancer, Quality of life, Epidemiology

## Abstract

**Study design:**

Systematic review.

**Objectives:**

Primary Spinal Intradural Tumours (PSITs) are rare pathologies that can significantly impact quality of life. This study aimed to review patient reported outcomes (PROs) in PSITs.

**Methods:**

A systematic search of Pubmed and Embase was performed to identify studies measuring PROs in adults with PSITs. PRO results were categorised as relating to Global, Physical, Social, or Mental health. Outcomes were summarised descriptively.

**Results:**

Following review of 2382 records, 11 studies were eligible for inclusion (737 patients). All studies assessed surgically treated patients. Schwannoma was the commonest pathology (*n* = 190). 7 studies measured PROs before and after surgery, the remainder assessed only post-operatively. For eight studies, PROs were obtained within 12 months of treatment. 21 PRO measurement tools were used across included studies, of which Euro-Qol-5D (*n* = 8) and the pain visual/numerical analogue scale (*n* = 5) were utilised most frequently. Although overall QoL is lower than healthy controls in PSITs, improvements following surgery were found in Extramedullary tumours (EMT) in overall physical, social, and mental health. Similar improvements were not significant across studies of Intramedullary tumours (IMT). Overall QoL and symptom burden was higher in IMT patients than in brain tumour patients. No studies evaluated the effect of chemotherapy or radiotherapy.

**Conclusion:**

Patients with PSITs suffer impaired PROs before and after surgery. This is particularly true for IMTs. PRO reporting in PSITs is hindered by a heterogeneity of reporting and varied measurement tools. This calls for the establishment of a standard set of PROs as well as the use of registries.

## Background

Primary Spinal Intradural Tumours (PSIT) include intramedullary (IMT) and extramedullary (EMT) tumours. IMTs are those that arise from the spinal cord itself and EMTs are those that arise from the structures within the dural sac but not from the spinal cord. Both IMTs and EMTs are rare tumours with an estimated incidence of 0.74 per 100,000 person years for both subtypes combined [1]. EMT tumours represent 70% of all intradural tumours, most of which are benign and amenable to surgical resection. A broad range of pathologies fall under the PSIT label, the commonest being meningiomas, schwannomas, and ependymomas [2]. IMTs in adults are most commonly ependymomas or astrocytomas. These are mostly slow growing, however 10% are more aggressive in their growth [3]. The mainstay of treatment for both EMTs and IMTs is early surgery [4]. However, surgical treatment for IMTs often involves performing a myelotomy with an increased risk of residual or worsened neurological deficit. As such, outcomes for IMTs may be worse than for EMTs.

PSITs may present with almost any neurological deficit affecting the upper or lower limbs, bladder or bowel dysfunction, or axial or radicular pain [5]. They therefore have the potential to substantially impact a patients functioning and quality of life. Patient Reported Outcomes (PROs) are measures coming directly from patients about a given health condition or treatment [6]. This umbrella term encompasses measures of health related quality of life (HRQoL), health status, symptom burden, adherence to treatment, and satisfaction with treatment [7]. Measurement of PROs is becoming increasingly important to planning and delivering clinical effectiveness research, for healthcare organisations, and for monitoring individual patient care [8]. The objective of this systematic review was to summarise all primary research that reports PROs in cohorts of adult patients with PSITs.

## Methods

The protocol for this review was registered on PROSPERO (CRD 42022290780) and reporting has been performed in accordance with the Preferred Reporting Items for Systematic Reviews and Meta-Analyses (PRISMA) guidelines 2020.

### Eligibility criteria

Eligibility criteria for study inclusion are listed in Table 1.

**Table 1:** Eligibility criteria for study inclusion.

Inclusion criteria	Exclusion criteria
Including PSITs collectively or EMT/IMT separately	Extradural or metastatic tumours
Reporting PROs for PSIT population	Cadaveric studies
	Non-human studies
	Including <10 patients
	Non-primary publication (e.g. reviews, editorials, guidelines, etc)

### Search strategy

Searches were performed in PubMed and Embase using a comprehensive search strategy (Supplementary Appendix 1). The searches were performed 05/11/2021. This search strategy was developed to identify the full breadth of PRO research involving PSITs. Finally, the reference lists for all included articles of this systematic review were examined for further eligible articles.

### Selection process

Removal of duplicate articles was performed using EndNote X9.3.3. Abstract screening was performed on the online systematic review management platform (Rayyan). Abstracts were divided such that each abstract was reviewed by at least two co-authors (MAM, OA, CG, GR, AMSA). Full texts for articles included at the end of the screening process were reviewed against the eligibility criteria. Reasons for exclusion of articles were recorded at the full text review stage. Where studies appeared to be from the same author group and/or based on the same patient population, the study with the latest publication date was used.

### Data collection process

Each article was read by two of five reviewers (AMSA, MAM, OA, CG, GR). All data were extracted and independently entered into a Microsoft Excel spreadsheet (v16.34, Microsoft, Washington DC, USA). Summary study details as well as details of PROs reported were extracted from articles. To facilitate analysis of PROs, these were categorised into global, physical, social, and mental health PROs.

Additional data extracted included: demographics and number of patients included: participation rate (the percentage of patients responding to invitations to contribute PROs), tumour histology, IMT/EMT location, treatment provided, method of PRO assessment (questionnaire vs. interview), study type, and timepoint of PRO assessment (e.g. before or after surgery). Where data was not obtainable, this was documented.

### Quality of reporting

Quality of reporting in each article was assessed according to a modified version of the International Society for Quality of Life Research (ISOQOL) reporting standards. Although originally designed for use with PROs reported in randomised clinical trials (RCT), the ISOQOL standards have been implemented in systematic reviews assessing a variety of study types [9]. However, the modified version permits the use of these standards for any study type (see Supplementary Appendix 2 for full details) [10]. This was performed by one of four data collectors (MAM, OA, CG, GR) and each data point independently checked by the first author (AMSA).

### Study risk of bias assessment

Two different tools were used to assess the risk of bias according to study type. The Joanna Briggs Institute (JBI) checklist was used for cross-sectional studies [11]. The Newcastle-Ottawa Scale (NOS) was used for cohort studies [11] The JBI and NOS tools were applied independently by one of four reviewers (MAM, OA, CG, GR) and each item independently confirmed by the first author (AMSA).

## Results

### Study selection

**Fig. 1:**
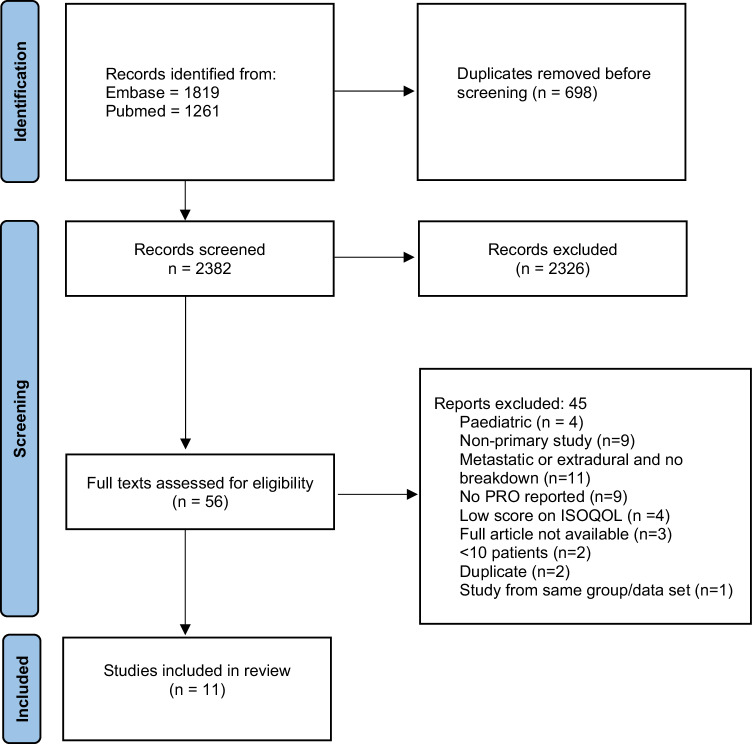
PRISMA flow diagram of search results. PRISMA flow diagram demonstrating search results, excluded studies, and included studies.

The literature search identified 2382 studies following removal of duplicates. Following abstract screening, 56 studies remained for full text review. A total of 11 studies were included in the final analysis. Figure 1 demonstrates this study’s PRISMA flowchart with reasons for exclusion after full text review.

### Study characteristics

Nearly half of included studies originated from North America (*n* = 5). The remainder were from Europe (*n* = 3), Japan (*n* = 2), and one study from South America. The total number of PSIT patients represented across these studies was 737 (range: 16–149). Of these, 392 were EMTs and 345 were IMTs. One study contained a control group in the form of brain tumour patients [12]. The commonest pathology was schwannoma (*n* = 190). The mean participation rate in these studies was 74% (range 43–97%). Where the mean age of the patients was reported, (*n* = 8 studies), the average age of all patients in this review was 44.6 years. Overall, 51.7% of patients were female.

Four studies were focused on EMT, five studies were on IMT, and two studies included both subtypes. In the two studies reporting on both pathological subtypes, outcomes were not broken down by EMT/IMT status. There were 4 cross sectional and 7 longitudinal cohorts. The longitudinal cohorts all assessed pre- and post-operative PROs. All studies were surgical series, and no study evaluated the impact of chemotherapy/radiotherapy. PRO assessments were collected through several different routes. This included completion by the patient online [12], in clinic [13–16], by mail [17–19], in person asked by an interviewer [20], via phone [21], or by a combination of methods [22]. For a full list with abbreviations of PRO tools used, see Table 2.

**Table 2:** Summary of study findings.

Study	No. of patients completing surveys	Tumour category	Pathology (n)	Assessment time points and follow up (f/u)	Overall reported health	Physical health	Social health	Mental health
Tarantino et al.	104	EMT	Schwannoma (51) Meningioma (31) Filum ependymoma (11) Neurofibroma (5) Other (6)	Pre-op and 12 months post-op	Overall post-op QoL rated as middle to high using VAS component of the EQ5D with a mean of 61.8 Worst QoL for sphincter disturbance (33.4), sensorimotor (43.3), and motor deficits (44.8) Both neurological deficits and pain were significant predictors of worse QoL. However, deficits presence and severity were a much stronger predictor of worsened QoL. Highest VAS QoL for patients with no deficits or sensory deficits only.	GRS pain reduction of 2.4 points at 12 months post-op. No significant effect of age, tumour type, or tumour location.	NA	NA
Viereck et al.	44	EMT	Schwannoma (26) Meningioma (14) Ependymoma (3) Neurofibroma (1)	Pre-op, and post-op <1 month, 1–3 months, and 3–12 months, and >12-months	NA	At < 1 month f/u, the ODI and the EQ-5D mobility domain were both significantly worse than pre-op. However, both demonstrate a trajectory of improvement at the 1-3, 3-12, and > 12 month f/u timepoints, reaching significant by 3-12 month f/u timepoints and beyond. EQ-5D-3L self-care domain demonstrated similar patterns to the above domain although was not significant at 12 months. VAS for pain and EQ-5D-3L pain/discomfort domain demonstrated significant improvement at all f/u post -op.	EQ-5D usual activities domain demonstrated improvement by the 3–12 months f/u point and remained significant to 12 months.	On the EQ-5D-3L Anxiety and Depression domain, 21 patients reported some, 6 severe, and 15 no anxiety or depression. Post-op this significantly improved compared to pre-op at 1–3 month and 3–12 month f/u. No difference was demonstrated at < 1 month and > 12 month f/u.
Chotai et al.	38	EMT	Schwannoma (15) Meningioma (10) Neurofibroma (8) Other (5)	Pre-op and 12 month post-op	Statistically significant improvement at 1 year in overall QoL assessed using the EQ-5D (mean of 0.62 vs. 0.77)	Statistically significant improvement at 1 year was found in disability (using the ODI/NDI), pain (using the NRS), and general physical function (using the SF36 PCS).	NA	Statistically significant improvement in mental health at 1 year (using the MCS component of the SF36)
Newman et al.	28	EMT	Schwannoma (35) Meningioma (18) Neurofibroma (2) Other (2) (All patient evaluated in the study, not all completed the surveys)	Pre-op and 6-, 12- and >12-month. Analysis using last f/u.	All BPI and MDASI scores statistically significantly improved at last f/u compared to pre-op. Greatest improvement for cervical location tumours.	Within the BPI, significant improvements found in pain, general activity, walking ability, and quality of sleep. Only non-significant change in the ‘relief’ item (assessing degree of relief using analgesics) All physical health domains of the MDASI showed significant improvement at last f/u.	Ability to participate in work and normal relations as assessed by the BPI and MDASI showed significant improvements at last f/u.	Overall mood and enjoyment of life as assessed by the BPI and MDASI showed significant improvement at last f/u.
Nakamura et al.	85	IMT	Ependymoma (43) Astrocytoma (17) Hemangioblastoma (13) Cavernous angioma (8) Other (4)	Post-op (mean f/u 5.4 years)	SF36 worse than national averages. Especially for mental and physical health. All SF36 domains negatively correlated with NPSI.	NPSI > 10 (moderate to severe pain) found in 56% of patients. Wide distribution of patients scoring between 10-50 compared to 0-10. No effect of age/gender. Paresthesiae/dysesthesia category of the NPSI statistically significantly worse than other pain categories (burning, pressing, or paroxysmal pain) High NPSI scores despite improved JOA scores associated with hemangioblastoma pathology. Burning pain associated with astrocytoma and paroxysmal pain associated with hemangioblastoma.	NA	NA
Nakanishi et al.	16	IMT	Ependymoma (16)	Pre-op and 6–12 months post-op	Across all domains of the SF36, no statistically significant change was found between pre- and post-op scores. This held true for patients with MMG 1–2, or 3–5 and no effect of tumour location was found. A statistically significant negative correlation was found between SF36 scores and MMG, such that a lower MMG was associated with a better SF36 score.	NA	NA	NA
Xiao et al.	45	IMT	Ependymoma (27) Hemangioblastoma (6) Astrocytoma (4) Cavernoma (3) Other (5)	Post-op 22 months f/u (median)	EQ-5D MCID was reached in 28% of patients. However, overall mean EQ-5D did not reach statistical significance post-op (0.627 vs. 0.608, *p* = 0.73) nor in any of its 5 domains. Improvement in post-op EQ-5D predicted by better pre-op EQ-5D, improved post-op neurological status (assessed using MMG). Syrinx formation and CSF leak predicted worsened EQ5D. Longer follow up trended towards improved EQ-5D. Predictors of reaching MCID in EQ-5D included: pre-op EQ-5D and MMG, and length of hospital stay. Ependymoma pathology trended towards predicting achieving MCID. Cervical location was associated with not reaching MCID.	PDQ MCID was reached in 28% of patients. However, no significant difference found between pre- and post-op PDQ scores (72.1 vs. 74.7, *p* = 0.76). Improvement in PDQ predicted by better post-op status on MMG and thoracic location. Worsening PDQ predicted by worse pre- and post-op neurological status on MMG, older age, post-op CSF leak, cervical and cervicothoracic locations. Ependymoma pathology and pre-op PDQ were predictors of reaching MCID in PDQ.	NA	PHQ-9 MCID was reached in 16% of patients. However, no significant difference was found between pre- and post-op PHQ-9 scores (7.76 vs. 7.11, p = 0.60). PHQ-9 improvement predicted by pre-op PHQ-9 score and length of hospital stay. No predictors of reaching MCID found for PHQ-9.
Acquaye et al.	149	IMT	Ependymoma (149) *Compared to brain ependymoma patients*	66 months f/u (median)	Overall higher symptom burden for spine tumour patients compared to brain tumour patients (measured using the MDASI – 2.8 vs. 1.9). Higher number of spine patients reporting symptoms (57% vs. 44%). Worse overall health and vitality reported using SF36 in spinal patients.	Commonest reported symptoms in spine tumour patients according to MDASI: numbness/tingling (59%), fatigue (52%), weakness (47%), pain (46%), and sexual dysfunction (39%). Worse PCS in SF36 in spine vs. brain tumour patients.	Greater impact on ability to work and engaging in activities reported by spine patients than by brain tumour patients.	Experiencing depression reported in 15% (compared to 19% in brain tumour patients)
Butenschoen et al.	65	IMT	Ependymomas (65)	5.4 years f/u (median)	High disease burden and perceived disability on the mean utility (mu) EQ5D (0.676). Significantly worse mu than EMT tumours and if not able to return to physical activities and for female patients. Overall quality of life worse with existence of neurological deficits and worsened MMG. No correlation with age.	Within SF36, lowest scores observed for vitality and role limitations due to physical constraints. Reduced SF36 in all categories in patients with difficulties returning to sports. Participation in individual sports remained stable (although reduced in session frequency per week), but reductions in team sports. Commonest limitations: pain, coordination problems, motor deficits, and fear of injury. Worsened sports participation predicted by MMG, laminectomy or laminoplasty (vs. unilateral laminotomy), and >5 segments being operated on.	Reduction in those continuing with full time work (52% to 32%). Increase in those shifting to part time work or retiring early. Commonest reasons: persistent pain and physical stress. No association found with spinal level, age, WHO grade, or gender.	NA
Bellut et al.	63	Both	Meningioma (23) Schwannoma (15) Ependymoma (13) Astrocytoma (2) Other (10)	Pre-op, 3- and 12- month post-op.	COMI overall score and QoL domain showed significant improvement at 3-months, and further significant improvement at 12-months. Overall satisfaction with surgery was high (assessed using a general satisfaction Likert scale). 95% satisfied or very satisfied at 3- and 12-month f/u. Overall feeling of surgery being helpful was high (assessed using a general Likert scale). 84% and 85% found surgery had helped or helped a lot at 3- and 12-months respectively.	Axial and peripheral pain domains of the COMI showed significant reduction at 3-months, sustained at 12-months. Further improvements at 12-months were found in the ‘worst pain’ category of the COMI.	Social disability domain of the COMI showed significant improvement at 3-months, with further improvement at 12-months. Work disability domain of the COMI only showed improvement at the 12-month f/u.	NA
Guirado et al.	100	Both	Schwannoma (33) Meningioma (24) Ependymoma (23) Cavernoma (5) Neurofibroma (4) Other (11)	Pre-op and post-op (mean f/u 20 months)	Higher SF36 score overall, the general health domain, and the vitality domain of the SF36 were significantly associated with an improved post-op neurological status (measured with the MMG and all subscales of the ALS (gait, micturition, bowels)).	SF36 PCS domain significantly associated with post-op myelopathy subscales: MMG (*p* < 0.001), gait ALS (*p* < 0.001), micturition ALS (*p* = 0.003), bowel ALS (*p* = 0.003). SF36 vitality domain significantly associated with bowel ALS (*p* = 0.013).	Social functioning domain of SF36 significantly associated with MMG (*p* < 0.001). Role functioning-emotional SF36 domain was associated with bowel ALS (*p* = 0.007).	Poorer MCS in the SF36 was associated with poorer ALS gait, micturition ALS (*p* = 0.042), and bowel ALS (*p* = 0.003). subscales.

*ALS* Aminoff-Logue Scale, *BPI* Brief Pain Index, *COMI* Core Outcome Measures Index, *EMT* Extra Medullary Tumour, *EQ5D* EuroQol 5 Dimensions, *GRS* Graphic Rating Scale (from 0-no pain to 10-severe pain), *IMT* Intra Medullary Tumour, *JOA* Japanese Orthopaedic Association score, *MCID* Minimal Clinically Important Difference, *MCS* Mental component score/summary, *MDASI* MD Anderson Symptom Inventory, *NDI* Neck Disability Index, *NPSI* Neuropathic Pain Symptom Inventory, *NRS* Numeric Rating Scale, *ODI* Oswestry Disability Index, *PCS* Physical component score/summary, *PDQ* Pain Disability Questionnaire, *PHQ-9* Patient Health Questionnaire 9, *QoL* Quality of Life, *SF36* Short Form 36, *VAS* Visual Analogue Scale. The following scale used is not a PRO: MMG – Modified McCormick Grade.

### Risk of bias in studies

The results for risk of bias assessments are presented in Supplementary Appendix 3.

### Quality of PRO reporting within studies

All studies included in this review achieved satisfactory scoring on the modified ISOQOL reporting standards. Due to heterogeneous reporting, varied levels of granularity, and varied PRO tools used, quantitative meta-analysis of findings was not possible. A qualitative synthesis of findings was therefore used.

#### Outcomes reported in EMTs

##### Global health

For EMTs, numerous studies identified an improvement in overall quality of life post-operatively at 12 months [13, 21] and beyond [22]. Post-operative overall QoL as measured using the Visual Analogue Scale (VAS) component on the EQ-5D was middle to high [13]. Although ongoing presence of pain was associated with worsened QoL, the strongest predictors of this were bladder/bowel symptoms and motor deficits [13]. Presence of sensory deficits alone did not significantly influence overall QoL [13]. Where studies attempted to identify the impact of tumour location, cervical tumours were associated with the greatest improvement post-operatively [22].

##### Physical health

In all EMT studies, significant improvement in post-operative pain at 12-months follow up were reported across a variety of reporting tools (Table 2) [13, 14, 21, 22]. This improvement occurred as early as <1-month follow-up [14] and was not associated with age, tumour type, or tumour location [13].

Physical functioning also improved post-operatively at 12-months measured using the Oswestry Disability Index (ODI), Neck Disability Index (NDI), or the Physical Component Score (PCS) component of the SF36 [21]. However, unlike the early improvement in pain reported above, an initial worsening in the ODI as well as the mobility and self-care EQ5D domains at <1-month was reported by Viereck et al, followed by improvement at all subsequent follow ups [14]. Assessed using the Brief Pain Index (BPI) and the MD Anderson Symptom Inventory (MDASI), Newman et al also reported significant improvements in physical health, general activity, walking ability, and sleep quality at >12-months [22].

##### Social health

Social health in EMTs was assessed in two studies. Participation in usual social activities as measured using the EQ5D appeared to significantly improve by 3–12 months (Table 2) [14]. This change was sustained beyond 12 months as measured using the BPI and MDASI [22].

##### Mental health

Self-reported depression and anxiety was measured using a variety of general tools including the EQ5D, SF36, BPI, and the MDASI (Table 2). In a cohort of 44 EMTs, Viereck et al reported a improvements in the anxiety and depression scores of the EQ5D post-operatively as early as <1-month, and at 1–3 months, 3-12 month, and > 12-month follow up [14]. Likewise, Chotai et al reported a significant improvement in overall mental health in a group of EMTs at the 12 month mark using the Mental Component Score (MCS) of the SF36 [21]. Overall mood and enjoyment of life also demonstrated significant improvement as measured using the BPI and MDASI at long term follow up [22].

#### Outcomes reported in IMTs

##### Global health

In contrast to EMTs, a general pattern of worsened overall QoL was reported for IMTs. Compared to national averages, overall SF36 scores were worse at a mean follow of 5.4 years post-operatively [17]. These were significantly associated with the existence of pain, as measured using the Neuropathic Pain Symptom Inventory (NPSI). In another cohort, although the minimal clinically important difference (MCID) in the EQ5D was reached in 28% of IMTs, the group average did not show a statistically significant improvement at a median of 12 months of follow up [16]. Ependymoma pathology was trending towards reaching MCID. In contrast to findings in EMTs by Newman et al, cervical location was associated with worsened outcomes in IMTs [22].

Three studies included ependymoma patients only. In a study of 16 ependymoma patients by Nakanashi et al. no improvement in total SF36 score post-operatively was identified at a follow up of 6–12 months [20]. At a median follow up of 5.4 years, Butenschoen et al noted an overall high disease burden and perceived disability in ependymoma patients as measured on the mean utility EQ5D [19]. A worsened outcome was associated with a worse neurological deficit as measured with the MMG, not being able to return to usual physical activities, and female gender. Acquaye et al compared ependymoma pathology in spinal versus cranial patients. They note a significantly higher symptom burden and symptom frequency in spinal ependymoma patients as well as overall worse SF36 scores as compared to cranial patients.

##### Physical health

In contast to EMTs, improvements in pain were not as prevelant in IMTs. At a mean follow-up of 5.4 years, 56% of 85 IMTs were found to have remaining moderate to severe pain measured using the NPSI [17]. The paresthesiae/dysesthesia domain of the NPSI was significantly worse than other pain categories (burning, pressing, or paroxysmal). Acquaye et al found the commonest reported symptom in ependymoma patients being numbness/tingling (reported by 59% of 149 patients) as well as pain (reported by 46%) [12]. Tumour pathology was found to potentially influence the type of pain perceived with astrocytomas being associated with burning pain and hemangioblastomas being associated with paroxysmal pain [17]. The worsening pain scores were independent of improvements in functional status, as measured using the Japanese Orthopoedic Association score (JOA).

For overall physical health, at 22 months median follow up, Xiao et al reported no significant improvement in mean Pain Disability Questionnaire (PDQ) scores despite 28% reaching MCID [16]. Improvements were associated with ependymoma pathology and thoracic location, while a worsening of the PDQ was associated with cervical location and the development of cerebrospinal fluid leak post-operatively. Acquaye et al reported that the PCS of the SF36 was worse in spinal ependymoma than cranial ependymoma patients [12]. They also identified specific physical symptoms that were reported frequently by ependymoma patients to include fatigue (46%), weakness (47%), and sexual dysfunction (39%). Butenschoen et al reported the worst SF36 domains for ependymoma patients being vitality and role limitations due to physical constraints [19]. These were significantly worse in patients reporting difficulties returning to any sporting activity.

##### Social health

Compared to brain ependymoma patients, spinal patients demonstrated reduced return to work and participation in usual activities [12]. There was a general shift from full time employment pre-op to part time or early retirement. Commonest reasons reported for this was persistent pain or physical stress. Individual level participation in sports remained stable, although overall frequency of sporting sessions reduced as well as a reduction in participation in group sports. Commonest reported limitations for participation in sports were pain, coordination problems, motor deficits, and a fear of injury. Poorer participation in sports was associated with poorer MMG post-operatively and more extensive operative approaches ( > 5 levels or laminectomy/laminoplasty vs. unilateral laminotomy).

##### Mental health

In a mixed group of IMTs [16], mean Patient Health Questionnaire 9 (PHQ-9) scores did not significantly differ between pre- and post-operative assessments (22-months median follow up). This was the only study where a dedicted mental health assessment tool was used. MCID in the PHQ-9 was reached in 16% of patients, but no predictors of improvement were identified. Compared to cranial ependymoma patients, the rates of self-reported depression were similar in spinal patients (19% vs. 15%) [12].

#### Outcomes reported in combined EMT/IMT cohorts

##### Global health

Two studies reported outcomes of EMTs and IMTs without reporting the outcomes for each pathology subgroup separately. In a mixed study of EMTs and IMTs, Bellut et al found that overall QoL was significantly improved with surgery at 3- and 12-month follow up [18]. They also identified significant satisfaction with surgery and a patient reported sense of surgery having been a useful intervention. In another combined series, Guirado et al reported a correlation with improved post-operative SF36 scores and post-operative neurological status as measuring using the MMG and the Aminoff-Logue Scale (ALS) [15]. Of note, in both studies where a mixed population is reported, the majority of pathologies were EMTs.

##### Physical health

At a mean of 20 months follow up, Guirado et al reported a worsened PCS component of the SF36 in a combined EMT/IMT cohort which was associated with myelopathy subscales of the ALS (gait, micturition, and bowel) and with poorer post-operative MMG. The SF36 vitality domain was significantly associated with the bowel domain of the ALS. However, in another combined series, Bellut et al reported improvements in axial and peripheral pain measured using the Core Outcome Measures Index (COMI) as early as 3 months follow up, and this was sustained at 12-months follow up [18].

##### Social health

In mixed IMT and EMT studies, overall social disability as measured using the COMI improved by 3 months [18], however work disability improvement was only detected at 12 months [18]. Overall improvement in social functioning as measured using the social functioning domain of the SF36 was associated with improved MMG post-operatively [15]. Prescence of bowel symptoms on the ALS was associated with a poorer role functioning score on the SF36 [15].

##### Mental health

In a mixed cohort of IMTs and EMTs, presence of myelopathic symptoms as measured using the ALS was associated with poorer scores in the MCS of the SF36.

## Discussion

Collecting and using PROs in the evaluation of interventions allows for clinical care with a patient centred focus [23]. In this systematic review of 11 studies of PROs in 737 PSIT patients, global and physical health were the main patient reported dimensions. Despite the breadth of symptoms suffered by spinal patients, mental and social health was not reported as frequently. For EMTs, an overall improvement in outcomes after surgery was identified. However, for IMTs, poorer or unchanged outcomes across all PRO domains were found. This review also identified substantial heterogeneity in the PRO reporting in the field. In total, 21 PRO tools were identified. All studies evaluated the impact of surgical intervention. Most studies evaluated PROs up to 12-months post-operatively with few studies extending beyond this time point. The different trajectories of EMTs and IMTs necessitate reporting of outcomes separately for each category.

Overall QoL in EMTs was improved due to surgical interventions [13, 21, 22]. However, a similar pattern was not convincingly found in IMTs with no significant improvement found in mean QoL scores post-operatively [16, 20]. Across both tumour subtypes, predictors of better outcomes included an improved pre-operative, or post-operative neurological status and ependymoma pathology in IMTs [16]. Myelopathic symptoms including bladder or bowel symptoms in particular were identified as predictors of worsened overall QoL [13, 15].

Pain was measured with a variety of tools including the VAS, GRS, NRS, BPI, and MDASI. Across all measures, studies in EMTs demonstrates significant improvement in pain post-operatively that was long-lasting into the 12-month follow-up range [13, 14, 21, 22]. In contrast however, chronic pain pain was more common in IMTs and was a significant contributor to worsened QoL [17]. Interestingly, although the sample sizes were small in this study by Nakamura et al, associations were found between pathology of IMT and the *subtype* of pain.

HRQoL is a multi-dimensional entity, recognised in the varied domains questioned in commonly utilised tools such as the EQ5D or the SF36. Spinal pathology in particular has the potential to severely affect HRQoL through its impact on motor, sensory, pain, bladder, bowel, or sexual function. With the potential to create such varied deficits, it is pertinent to evaluate the effect of interventions on patient return to work and usual social activities that can improve quality of life such as engagement in family time or sporting activities. Few studies directly evaluated such dimensions. Where assessed, in EMTs a return to usual activities had significantly increased by 3 months [14] and sustained up to 12 months [22]. In contrast, for ependmomas, there was a reduction in return to work [19] and engagement in usual activities compared to brain ependymoma patients [12]. More invasive surgical approaches were associated with a reduction in patient return to sporting activity [19].

Although not often reported, improvements in mental health were sustained up to the 12-month mark in EMTs [14, 21]. The only dedicated depression/anxiety assessment tool used was the PHQ-9 in a cohort of IMTs where no significant change post-operatively was detected [16]. Compared to patients with brain ependymomas, the rates of self-reported depression were not significantly different to spinal patients (19% in brain, 15% in spine ependymoma patients) [12]. It is likely that with the significant symptom burden identified in this review and the impact on physical and social health that a significant mental health burden also exists. Regular collection of such outcomes is therefore pertinent to highlight the broader needs of this patient group.

In total, 21 PRO tools were used across studies. The SF36 was the commonest tool used. While the SF36 has been validated for other neurological [24] and non-neurological [25] conditions, it has not been validated for use in PSITs. However, the use of such global tools as the SF36 and EQ5D allow assessment of a broad range of HRQoL domains and comparison with other conditions. To allow the quantitative comparison of outcomes, a standardised set of PRO tools to be used in future reporting in spinal pathology is needed. In addition, use of tools with validated MCID would allow the identification of variables that lead to this clinically meaningful result. PRO tools tailored to account for the complex and varied deficits in spinal patients must also be used [15].

Acquaye et al compared ependymoma pathology in cranial and spinal patients and identified crucial differences between the two groups [12]. This included higher overall symptom burden in spinal patients, worse overall QoL, vitality scores, and PCS scores in the SF36, poorer return to work and usual activities in spinal ependymoma patients as well as comparable rates of depression and anxiety. These findings highlight the severity of symptoms suffered by patients with IMTs.

Finally, there is an emerging recognition that radiotherapy and chemotherapy have substantial effects on neural tissue as displayed in the reduction in cognitive function for patients receiving radiotherapy to the brain [26] or chemotherapy for any type of cancer [27]. However, no studies reported the effects of any such therapies. It is therefore difficult to disentangle the effects of surgery from those of chemotherapy/radiotherapy. This is particularly true for IMTs, where overall poorer outcomes were found and where chemotherapy or radiotherapy are more likely to be indicated [28]. Evaluating the impact of chemotherapy and radiotherapy is certainly an area for future focus in the field and again calls for the creation of granular registries.

## Conclusion

HRQoL as measured by PROs demonstrated significant and long-lasting overall improvement in patients with EMTs following surgical intervention. This improvement extended to improvements in pain, physical health, social function, and mental health. Similar improvements in IMTs were not found. No studies were found that evaluated the impact of chemotherapy or radiotherapy on patients with PSITs. Also, there exists substantial heterogeneity in the literature reporting of PROs in PSITs. This heterogeneity precludes the quantitative evaluation of the effect of tumour pathology, location, and surgical approach – key variables needed in counselling and planning care for patients. This calls for the establishment of a key set of PROs for this patient group and possibly the use of granular registries to allow the pooling of data on these rare pathologies.

### Supplementary information

Supplementary material 1 - Search strategy

Supplementary material 2 - ISOQOL

Supplementary material 3 - Risk of bias assessment
