# Supplementary material for: Patient-reported outcomes in Primary Spinal Intradural Tumours: a systematic review
Source: Spinal Cord. 2024 Apr 8;62(6):275–84. doi: 10.1038/s41393-024-00987-6 (PMC11199135; doi:10.1038/s41393-024-00987-6)
Supplement: Supplementary file 1 — Supplementary material 1 - Search strategy [file 41393_2024_987_MOESM1_ESM.pdf]

Patient reported outcomes in Primary Spinal Intradural Tumours: a systematic review (PROSIT-SR)

**Database**: PubMed		
**Date: 10/11/2021**		
Number	Term	Results
#1	Spine[tiab] OR spinal[tiab] OR cervical[tiab] OR thoracic[tiab] OR lumbar[tiab] OR sacral[tiab] OR filum terminale[tiab] OR vertebr*[tiab]	930,475
#2	Intramedullary[tiab] OR intradural[tiab] OR extramedullary[tiab] OR intraspinal[tiab] OR cord[tiab]	267,254
#3	Tumour*[tiab] OR tumor*[tiab] OR neoplas*[tiab] OR meningioma*[tiab] OR schwannoma*[tiab] OR nerve sheath[tiab] OR ependymoma*[tiab] OR glioma[tiab] OR astrocytoma[tiab] OR dumbbell[tiab] OR neurofibroma*[tiab] OR Ganglioglioma[tiab] OR haemangioblastoma[tiab] OR lymphoma[tiab]	2,387,874
#4	#1 AND #2 AND #3	17,941
#5	Spinal Cord Neoplasms[MESH]	11,468
#6	Spinal Neoplasms[MESH]	14,897
#7	Spinal meningioma[Mesh:NoExp]	20,947
#8	Spinal meningeal neoplasms[Mesh:NoExp]	18,295
#9	#4 OR #5 OR #6 OR #7 OR #8	60,231
#10	Patient Reported Outcome Measures[Mesh]	12,507
#11	patient reported[tiab] OR patient rated[tiab] OR patient assessed[tiab] OR patient evaluated[tiab] OR self reported[tiab] OR self rated[tiab] OR self assessed[tiab] OR self evaluated[tiab] OR person reported[tiab] OR person rated[tiab]	260,661
#12	PROM[tiab] OR PROMs[tiab]	7,197
#13	Quality of Life[Mesh:NoExp]	252,607
#14	quality of life[tiab]	343,395
#15	QOL[tiab]	49,369
#16	HRQOL[tiab]	22,280
#17	HRQL[tiab]	3,988
#18	anxiety[tiab]	246,288
#19	depression[tiab]	406,018
#20	fatigue[tiab]	117,789
#21	#10 OR #11 OR #12 OR #13 OR #14 OR #15 OR #16 OR #17 OR #18 OR #19 OR #20	1,185,825
#22	#9 AND #21	1,702
#23	**#22**<br>Filters applied: Humans, English, from 1990 -	1,261

**Database**: EMBASE		
**Date: 10/11/2021**		
Number	Term	Results
#1	(Spine OR spinal OR cervical OR thoracic OR lumbar OR sacral OR filum terminale OR vertebr*).ti,ab	1184516
#2	(Intramedullary OR intradural OR extramedullary OR intraspinal OR cord).ti,ab	335734
#3	(Tumour* OR tumor* OR neoplas* OR meningioma* OR schwannoma* OR nerve sheath OR ependymoma* OR glioma OR astrocytoma OR dumbbell OR neurofibroma* OR Ganglioglioma OR haemangioblastoma OR lymphoma).ti,ab	3023085
#4	#1 AND #2 AND #3	23039
#5	Exp ‘spinal cord tumor’/	15799
#6	Exp ‘spine tumour’/	12112
#7	#4 OR #5 OR #6	42886
#8	Exp ‘patient-reported outcome’/	45585
#9	(patient reported OR patient rated OR patient assessed OR patient evaluated OR self reported OR self rated OR self assessed OR self evaluated OR person reported OR person rated).ti,ab	276199
#10	(PROM OR PROMs).ti,ab	10550
#11	*"quality of life"/	129411
#12	(quality of life).ti,ab	546653
#13	(QOL).ti,ab	91281
#14	(HRQOL).ti,ab	34168
#15	(HRQL).ti,ab	6794
#16	(Anxiety).ti,ab	339529
#17	(Depression).ti,ab	539908
#18	(Fatigue).ti,ab	186252
#19	#8 OR #9 OR #10 OR #11 OR #12 OR #13 OR #14 OR #15 OR #16 OR #17 OR #18	1558156
#20	#7 AND #19	2160
#21	#20 AND [humans]/lim AND [english]/lim AND [1990-]	1819
