# Supplementary material for: Patient-reported outcomes in Primary Spinal Intradural Tumours: a systematic review
Source: Spinal Cord. 2024 Apr 8;62(6):275–84. doi: 10.1038/s41393-024-00987-6 (PMC11199135; doi:10.1038/s41393-024-00987-6)
Supplement: Supplementary file 2 — Supplementary material 2 - ISOQOL [file 41393_2024_987_MOESM2_ESM.docx]

**ISOQOL reporting standards used**

The original ISOQOL standards contain 17 core recommendations for all studies which report PROs. A further 11 recommendations are provided for studies where PRO is a primary outcome. Dirven et al. applied these standards to neuro-oncology RCTs and added four new items (4, 16, 19, 21) and split one item (into recommendations 11 and 12). Therefore, the final standards used in this study contained 23 recommendations for all PRO studies and 11 additional recommendations where PRO was a primary outcome. As these recommendations were developed for RCTs, not all recommendations were applicable to other study types, therefore it was possible for studies to receive ‘N/A’ for items. In line with previous systematic reviews, studies meeting 66% of the recommendations applicable to that given study type were deemed as having satisfactory reporting quality.

**Notes**

^a^ = Added by Dirven et al

^b^ = Originally one but split by Dirven et al

| **Number** | **Core Recommendation** | **Options** |
| --- | --- | --- |
| 1 | The PRO should be identified as an outcome in the abstract | YES NO |
| 2 | The PRO hypothesis should be stated and should specify the relevant PRO domain(s) if applicable | YES NO  N/A (if explorative study) |
| 3 | The mode of administration of the PRO tool and the methods of collecting data (e.g., telephone, other) should be described | YES NO |
| 4 ^a^ | Electronic modes of distribution? Referring to internet or tablet based administration  If Q3 is NO - this is a N/A | YES NO  N/A |
| 5 | The rationale for choice of the PRO instrument used should be provided | YES NO |
| 6 | Evidence of PRO instrument validity and reliability should be provided or cited. N/A if interview | YES NO |
| 7 | The intended HRQL data collection schedule should be provided  Or if cross sectional - study authors should say when PRO data was collected. | YES NO  N/A |
| 8a | PROs should be identified in the trial protocol | YES NO  N/A (if not a trial/ no trial protocol available) |
| 8b | Post hoc analyses should be identified | YES NO  N/A (if no post hoc analyses completed/declared) |
| 9 | The status of PRO as either a primary or secondary outcome should be stated | YES NO  N/A (if PRO only outcome of the study) |
| 10 | There should be evidence of appropriate statistical analysis and tests of statistical significance for **each PRO hypothesis tested** | YES NO  N/A (if no hypothesis) |
| 11 ^b^ | Extent of missing data should be stated | YES NO  N/A (if interview is the only method of PRO acquisition) |
| 12 ^b^ | Statistical approaches for dealing with missing data should be explicitly stated, and the extent of missing data should be stated | YES NO  N/A (if no missing data declared) |
| 13 | A flow diagram **or a description** of the allocation of participants and those lost to follow-up should be provided for PROs specifically | YES NO |
| 14 | The reasons for missing data should be explained | YES NO  N/A (if no missing data/ none declared) |
| 15 | The study patients’ characteristics should be described, including baseline PRO scores | YES NO |
| 16 ^a^ | Are PRO outcomes also reported in a graphical format? | YES NO |
| 17 | The limitations of the **PRO components** of the trial should be explicitly discussed.  This statement applies to both trials and other study designs | YES NO |
| 18 | Generalizability issues **uniquely related** to the PRO results should be discussed, if applicable. Do the authors explain whether their PRO results are generalisable? | YES NO |
| 19 ^a^ | Are PRO interpreted? (Not only restated)  Do the authors explain their findings/ put in context of other literature? | YES NO |
| 20 | The clinical significance of the PRO findings should be discussed. | YES NO |
| 21 ^a^ | Methodology used to assess clinical significance (in case this was addressed) | Yes NO  N/A (if NO to Q20) |
| 22 | The PRO results should be discussed in the context of the other clinical trial outcomes | YES NO  N/A (if PRO only outcome) |

| **Number** | **Additional Recommendation if stated PRO is primary outcome/ or only outcome** | **Options** |
| --- | --- | --- |
| 23 | The title of the paper should be explicit as to the inclusion of a PRO | YES NO |
| 24 | The introduction should contain a summary of PRO research that is relevant to the RCT | YES NO |
| 25 | Additional details regarding the hypothesis should be provided, including the rationale for the selected domain(s), the expected direction(s) of change, and the time points for assessment | YES NO  N/A (if no hypothesis) |
| 26 | A citation for the original development of the PRO instrument should be provided | YES NO |
| 27 | Windows for valid PRO responses should be specified and justified as being appropriate for the clinical context | YES NO |
| 28 | There should be a power/sample size calculation relevant to the PRO based on a clinical rationale (e.g., anticipated effect size) | YES NO  N/A |
| 29 | The manner in which multiple comparisons have been addressed should be provided | YES NO  N/A (if no reference to multiple comparisons) |
| 30 | The analysis of PRO data should account for survival differences between treatment groups if relevant | YES NO N/A  N/A (if no survival outcomes reported) |
| 31 | Results should be reported for all PRO domains (if multi-dimensional) and items identified by the reference instrument (i.e., not just those that are statistically significant) | YES NO |
| 32 | The proportion of patients achieving pre- defined responder definitions should be provided where relevant | YES NO  N/A |
| 33 | A copy of the instrument should be included if it has not been published previously | YES NO  N/A |
