# Supplementary material for: Patient-reported outcomes in Primary Spinal Intradural Tumours: a systematic review
Source: Spinal Cord. 2024 Apr 8;62(6):275–84. doi: 10.1038/s41393-024-00987-6 (PMC11199135; doi:10.1038/s41393-024-00987-6)
Supplement: Supplementary file 3 — Supplementary material 3 - Risk of bias assessment [file 41393_2024_987_MOESM3_ESM.docx]

**Risk of bias assessment**

| Reference | Tool used | Rating |
| --- | --- | --- |
| Tarantino et al | NOS | \| Selection \| Comparability \| Outcome \| \| --- \| --- \| --- \| \| *** \| * \| ** \| |
| Viereck et al | NOS | \| Selection \| Comparability \| Outcome \| \| --- \| --- \| --- \| \| *** \| * \| ** \| |
| Chotai et al | NOS | \| Selection \| Comparability \| Outcome \| \| --- \| --- \| --- \| \| *** \| * \| ** \| |
| Newman et al | NOS | \| Selection \| Comparability \| Outcome \| \| --- \| --- \| --- \| \| *** \| - \| ** \| |
| Nakamura et al | JBI | 1)Yes 2)Yes 3)Yes 4)No 5)Yes 6)Yes 7)Yes 8)Yes |
| Nakanishi et al | NOS | \| Selection \| Comparability \| Outcome \| \| --- \| --- \| --- \| \| *** \| * \| ** \| |
| Xiao et al | JBI | 1)Yes 2)Yes 3)Yes 4)Yes 5)Yes 6)Yes 7)Yes 8)Yes |
| Acquaye et al | JBI | 1)Yes 2)Yes 3)Yes 4)No 5)Yes 6)Yes 7)Yes 8)Yes |
| Butenschoen et al | JBI | 1)Yes 2)Yes 3)Yes 4)No 5)Yes 6)Yes 7)Yes 8)Yes |
| Bellut et al | NOS | \| Selection \| Comparability \| Outcome \| \| --- \| --- \| --- \| \| *** \| - \| * \| |
| Guirado et al | NOS | \| Selection \| Comparability \| Outcome \| \| --- \| --- \| --- \| \| *** \| * \| ** \| |

NOS – Newcastle-Ottawa Scale risk of bias checklist for cohort studies

*All cohort studies score ‘N/A’ for NOS ‘Outcome’ item 1 (Assessment of Outcome) as PROs are, by definition, self-reported and all studies would therefore obtain 0 stars for this item.*

JBI – Joanna Briggs Institute risk of bias checklist for cross-sectional studies.

*Checklist includes:*

1. *Were the criteria for inclusion in the sample clearly defined?*
2. *Were the study subjects and the setting described in detail?*
3. *Was the exposure measured in a valid and reliable way?*
4. *Were objectives, standard criteria used for measurement of the condition?*
5. *Were confounding factors identified?*
6. *Were strategies to deal with confounding factors stated?*
7. *Were the outcomes measured in a valid and reliable way (e.g. validated PROM)?*
8. *Was appropriate statistical analysis used?*
